# Mitochondrial modulation-induced activation of vagal sensory neuronal subsets by antimycin A, but not CCCP or rotenone, correlates with mitochondrial superoxide production

**DOI:** 10.1371/journal.pone.0197106

**Published:** 2018-05-07

**Authors:** Katherine R. Stanford, Thomas E. Taylor-Clark

**Affiliations:** Department of Molecular Pharmacology & Physiology, Morsani College of Medicine, University of South Florida, Tampa, FL, United States of America; Cinvestav-IPN, MEXICO

## Abstract

Inflammation causes nociceptive sensory neuron activation, evoking debilitating symptoms and reflexes. Inflammatory signaling pathways are capable of modulating mitochondrial function, resulting in reactive oxygen species (ROS) production, mitochondrial depolarization and calcium release. Previously we showed that mitochondrial modulation with antimycin A, a complex III inhibitor, selectively stimulated nociceptive bronchopulmonary C-fibers via the activation of transient receptor potential (TRP) ankyrin 1 (A1) and vanilloid 1 (V1) cation channels. TRPA1 is ROS-sensitive, but there is little evidence that TRPV1 is activated by ROS. Here, we used dual imaging of dissociated vagal neurons to investigate the correlation of mitochondrial superoxide production (mitoSOX) or mitochondrial depolarization (JC-1) with cytosolic calcium (Fura-2AM), following mitochondrial modulation by antimycin A, rotenone (complex I inhibitor) and carbonyl cyanide m-chlorophenyl hydrazone (CCCP, mitochondrial uncoupling agent). Mitochondrial modulation by all agents selectively increased cytosolic calcium in a subset of TRPA1/TRPV1-expressing (A1/V1+) neurons. There was a significant correlation between antimycin A-induced calcium responses and mitochondrial superoxide in wild-type ‘responding’ A1/V1+ neurons, which was eliminated in TRPA1^-/-^ neurons, but not TRPV1^-/-^ neurons. Nevertheless, antimycin A-induced superoxide production did not always increase calcium in A1/V1+ neurons, suggesting a critical role of an unknown factor. CCCP caused both superoxide production and mitochondrial depolarization but neither correlated with calcium fluxes in A1/V1+ neurons. Rotenone-induced calcium responses in ‘responding’ A1/V1+ neurons correlated with mitochondrial depolarization but not superoxide production. Our data are consistent with the hypothesis that mitochondrial dysfunction causes calcium fluxes in a subset of A1/V1+ neurons via ROS-dependent and ROS-independent mechanisms.

## 1. Introduction

The activation of nociceptive sensory nerves by noxious stimuli evokes defensive reflexes and pain, which serves to protect the body from further damage. Inflammation, infection, physical damage and exogenous irritants are capable of significant nociceptor activation. As such, excessive activation of this sensory subpopulation contributes to a wide-range of disease states including chronic pain, irritable bowel disease, colitis, arthritis and asthma [1,2]. Nociceptive sensory nerves are selectively activated by noxious stimuli due to their expression of a variety of receptors, including the allyl isothiocyanate (AITC)-sensitive transient receptor potential (TRP) ankyrin 1 (A1) channel and the capsaicin-sensitive TRP vanilloid 1 (TRPV1) channel [3,4]. Both TRPA1 and TRPV1 are nonspecific cation channels whose activation causes neuronal depolarization and action potential discharge. TRPV1 is expressed on the majority of nociceptive neurons [5–7], although it should be noted that some specific subsets of nociceptive Aδ- and C-fiber nociceptors lack TRPV1 [8–10]. TRPA1 is expressed on approximately 66% of TRPV1-expressing neurons and <10% of neurons lacking TRPV1 expression [5–7,11,12]. TRPA1 is activated by a wide variety of electrophilic compounds, including reactive oxygen species (ROS) and environmental pollutants [13–16]. TRPV1 is activated directly by capsaicin, extracellular protons and heat, and indirectly downstream of second messenger signaling [3,17].

One potential way in which inflammation may cause nociceptor activation is by inducing the dysfunction of mitochondria, which are densely packed within sensory nerve terminals [18,19]. Mitochondrial dysfunction encompasses events including morphology changes, ROS production, mitochondrial depolarization and calcium release. While catastrophic mitochondrial dysfunction induces apoptosis, mitochondrial dysfunction also initiates signaling events involved in cellular stress responses. Chronic inflammation induces changes in mitochondrial morphology associated with dysfunction [20], and there are reports of an increased rate of mitochondrial DNA mutations and altered activity of ROS scavengers such as glutathione and superoxide dismutase in inflammatory states [21–23]. Inflammatory signaling has been shown to directly inhibit complexes I, II, and IV of the mitochondrial electron transport chain (mETC), resulting in ROS production and mitochondrial depolarization [24–27].

We have previously demonstrated that mitochondrial dysfunction induced by antimycin A, an mETC complex III inhibitor, activates vagal sensory neurons and bronchopulmonary C-fibers via the gating of TRPA1 and TRPV1 [28]. Inhibition of complex III by antimycin A induces ROS production and mitochondrial depolarization [29,30], but the role of these events in mitochondrial dysfunction-induced neuronal activation has not been established. Here we investigated the influence of mitochondrial dysfunction on the activation of vagal sensory neurons using three mitochondrial agents: antimycin A, carbonyl cyanide m-chlorophenyl hydrazone (CCCP, mitochondrial uncoupling agent), and rotenone (mETC complex I inhibitor). Using live cell co-imaging, we simultaneously monitored cytosolic calcium (as a surrogate for neuronal activation) and either mitochondrial superoxide production or changes in mitochondrial polarization. We found that the three agents activated (i.e. caused significant calcium fluxes) only a subset of A1/V1+ neurons, yet there was no difference in mitochondrial superoxide production or mitochondrial depolarization between ‘responding’ and ‘non-responding’ A1/V1+ neurons. Nevertheless, we observed a significant correlation between antimycin A-induced calcium responses and mitochondrial superoxide production in wild-type ‘responding’ A1/V1+ neurons, which was eliminated in TRPA1^-/-^ neurons, but not TRPV1^-/-^ neurons. CCCP-induced calcium responses did not correlate with either superoxide production or mitochondrial depolarization. Rotenone-induced calcium responses correlated with mitochondrial depolarization but not superoxide production. Our data are consistent with the hypothesis that mitochondrial dysfunction causes the activation of a subset of A1/V1+ neurons via ROS-dependent and ROS-independent mechanisms.

## 2. Experimental procedures

### 2.1 Mouse models

Female TRPV1^-/-^ mice were mated with male TRPV1^-/-^ mice. Female TRPA1^-/-^ mice were mated with male TRPA1^+/-^ mice. Genotype of the offspring was confirmed using polymerase chain reaction. Wild type C57BL/6J mice were purchased from The Jackson Laboratory. All experiments were performed with approval from the University of South Florida Institutional Animal Care and Use Committee (AAALAC #000434).

### 2.2 Neuronal dissociation

Male 6–12-week-old mice were euthanized by CO_2_ asphyxiation followed by exsanguination. Vagal ganglia were isolated followed by an enzymatic and mechanic dissociation then individual neurons were plated onto poly-D-lysine and laminin coated coverslips as previously described [28]. Neurons were incubated at 37°C in antibiotic-free L-15 media supplemented with 10% fetal bovine serum and used within 24 hours.

### 2.3 Live cell imaging

Cells were loaded with 4μM Fura-2AM for 45–60 minutes at 37°C. During the last 15 minutes of incubation, 2μM 5,5′,6,6′-Tetrachloro-1,1′,3,3′-tetraethyl-imidacarbocyanine iodide (JC-1), or 5μM MitoSOX Red was added in some studies. Coverslips were loaded into a chamber and perfused with heated (33–34°C) 10mM HEPES buffer (154mM NaCl, 4.7mM KCl, 1.2mM MgCl_2_, 2.5mM CaCl_2_, 5.6mM D-glucose). All drugs were diluted in HEPES buffer. Concentrations of mitochondrial modulators (antimycin A, 10μM; CCCP, 10μM; rotenone, 5μM) which induce acute mitochondrial ROS production and mitochondrial depolarization without significantly effecting cellular viability were used [31]. The vehicle (dimethyl sulfoxide (DMSO), 0.1%) for these experiments was the highest concentration of DMSO used in the dilution of mitochondrial inhibitor stocks into HEPES buffer. For the majority of the studies, the mitochondrial modulators were followed by a combined challenge of 100μM AITC & 1μM capsaicin to identify neurons that express either TRPA1 or TRPV1 channels. Sub-millimolar AITC is a selective agonist of TRPA1 [4,11,32]; capsaicin is a selective agonist of TRPV1 [3,11,33]. Lastly, cells were challenged with 75mM KCl to facilitate identification of neurons and 5μM ionomycin (calcium positive control). In one set of experiments, neurons from TRPV1^-/-^ mice were exposed to antimycin A (10μM), followed by H_2_O_2_ (300μM) and AITC (100μM), prior to KCl (75mM) and ionomycin (5μM). In another set of experiments, neurons were perfused with 10mM HEPES buffer with a calculated [Ca^2+^]_free_ of 75nM (154mM NaCl, 1mM KCl, 1.2mM MgCl_2_, 0.71mM CaCl_2_, 2mM EGTA, 5.6mM D-glucose) for 1 minute prior to, during, and for 2 minutes after exposure to mitochondrial modulators (antimycin A, 10μM; CCCP, 10μM; rotenone, 5μM) or vehicle (0.1% DMSO). Neurons were then perfused with normal 10mM HEPES (with 2.5mM CaCl_2_) for 5 minutes prior to a combined challenge of 100μM AITC & 1μM capsaicin, then KCl (75mM) and ionomycin (5μM).

Images were taken every six seconds using microscopy (CoolSnap HQ2; Photometrics, Surrey, BC). Fura-2AM imaging (intracellular calcium concentration or [Ca^2+^]_i_) was monitored by sequential excitation at 340 and 380nm (emission at 510nm) followed in most studies by either excitation at 470nm (emission at 525nm and 610nm) for mitochondrial polarization (JC-1), or 535nm (emission at 610nm) for superoxide production (MitoSOX).

### 2.4 Imaging analysis

Images were analyzed using Nikon Elements (Nikon, Melville, NY). Each individual neuron was analyzed separately using a region of interest (ROI) that encompassed the entire intracellular region. If required, ROIs were tracked over time to account for any cellular movement. For each imaging method (Fura-2AM, MitoSOX, and JC-1), ROIs with an unstable baseline, noisy baseline or insufficient loading were eliminated from the analysis. For Fura-2AM imaging, cells which failed to exhibit an increase in [Ca^2+^]_i_ to both AITC & capsaicin and KCl challenges (> 30% the ionomycin maximal response) were eliminated. Additionally, neurons that possessed an initial JC-1 red/green (525nm/610nm) ratio under 0.5 were eliminated as this indicated mitochondrial depolarization prior to the start of the experiment. For statistical analyses, we analyzed the maximal Fura-2AM, JC-1 and MitoSOX responses during the three-minute drug treatment (see below for calculations).

Relative changes in [Ca^2+^]_i_ were determined ratiometrically (R) using Fura-2 fluorescence: 340nm/380nm. As such the impact of variations in Fura-2AM loading are negated. The average calcium response to the combined AITC and capsaicin was used to classify A1/V1+ vs. A1-V1- neurons: A1/V1+ neurons had an R_AITC/Caps_ > (R_bl_ + 2*SD_bl_), where R_AITC/Caps_ is the average 340/380 ratio during AITC/capsaicin treatment, R_bl_ is the baseline 340/380 ratio prior to treatment, and SD_bl_ is the standard deviation of R_bl_. In the time series analyses, responses are presented as the change of the 340/380 ratio (ΔR = R_1_—R_0_). In the bar graphs, scatterplots and statistical analyses, the response to mitochondrial modulation was determined using the maximal Δ340/380 (ΔR_max_ = Rmax—R_0_) for each neuron. An A1/V1+ neuron was defined as ‘responding’ to a particular mitochondrial agent if the [Ca^2+^]_i_ ΔR_max_ was greater than 3 * SD of the ΔR_max_ of A1-V1- neurons: ΔR_max(A1/V1+)_ > ΔR_max(A1-V1-)_ + 3 * SD_(A1-V1-)_ where ΔR_max(A1/V1+)_ is the maximal response to mitochondrial modulation in an individual A1/V1+ neuron, ΔR_max(A1-V1-)_ is the average maximal response in A1-V1- neurons, and SD_(A1-V1-)_ is the standard deviation of ΔR_max(A1-V1-)_. We have evaluated the effect of Fura-2AM loading on the calcium responses to mitochondrial modulation in these experiments and determined that there is no correlation between Fura-2 intensity at 380nm at baseline, an indicator of Fura-2AM loading, and the magnitude of ΔR_max_ (data not shown). This suggests that the effects of calcium buffering by Fura-2 are negligible in the dissociated vagal neurons used in these studies.

Mitochondrial superoxide production was analyzed using MitoSOX fluorescence (F, excitation at 535nm). Given that the reaction of superoxide with MitoSOX red is irreversible and that baseline physiologic ROS production by mitochondria will vary between individual neurons, we have chosen to correct for the slope in the time series studies using F_1(adjusted)_ = F_1_ - (S_0_ * t), where F_1_ is the raw fluorescence at a given timepoint (in arbitrary fluorescent units), S_0_ is the slope of the baseline (arbitrary fluorescent units/s) and t is time of the F_1_ measurement (in seconds). As MitoSOX loading varies between individual neurons, we have normalized the slope corrected data: F_1(adjusted)_/F_0(adjusted)_, where F_1(adjusted)_ is the slope corrected fluorescence at a given timepoint and F_0(adjusted)_ is the average of the slope corrected baseline. In order to determine the percentage of neurons that exhibited meaningful mitochondrial superoxide production compared to vehicle, we calculated the percentage of neurons whose individual maximal F_1(adjusted)_/F_0(adjusted)_ was greater than the average maximal F_1(adjusted)_/F_0(adjusted)_ for vehicle treatment + 3 * standard deviation of the maximal F_1(adjusted)_/F_0(adjusted)_ for vehicle treatment.

Mitochondrial polarization was determined using JC-1 fluorescence at both the green (525nm, which measures the monomeric form of JC-1) and red (610nm, which measures the aggregated form of JC-1) emission wavelengths. JC-1 accumulates in negatively charged mitochondria and forms ‘red’ aggregates. Upon mitochondrial depolarization, JC-1 disperses into the cytosol as the ‘green’ monomeric form. As mentioned above, the 525nm/610nm ratio was used to identify neurons with depolarized mitochondria at baseline. However, we chose to determine the effect of mitochondrial modulation on mitochondrial polarization using only the ‘green’ monomeric form (F, 525nm fluorescence), as JC-1 fluorescence of ‘red’ aggregates is susceptible to quenching, prone to oxidation and requires ~90 minutes to reach equilibrium (versus ~15 minutes for monomers) [34]. Data were normalized (F_1_/F_0_) where F_1_ is the emission fluorescence at 610nm at a given time point and F_0_ is the baseline emission fluorescence at 610nm. This normalization process allowed for the calculation of the relative change of mitochondrial membrane potential independent of baseline polarization and JC-1 loading. In order to determine the percentage of neurons that exhibited meaningful mitochondrial depolarization compared to vehicle, we calculated the percentage of neurons whose individual maximal F_1_/F_0_ was greater than the average maximal F_1_/F_0_ for vehicle treatment + 3 * standard deviation of the maximal F_1_/F_0_ for vehicle treatment.

### 2.5 Statistical analyses

Statistical analyses were performed using R studio using maximal responses of the normalized responses observed during the three-minute drug treatment. To determine statistical differences in calcium response, mitochondrial polarization, and ROS production between mitochondrial agents and specific neuronal populations we performed two-way ANOVAs followed by post hoc comparison using the Bonferroni correction. To evaluate the correlation between cytosolic calcium and ROS production/mitochondrial depolarization, we performed a one-tailed Pearson correlation. A chi square test for homogeneity was used to evaluate the percentage of responding neurons for each inhibitor. A p value of 0.05 was taken as the threshold for significance.

### 2.6 Chemicals

Fura-2AM was purchased from TEFLabs (Austin, TX). MitoSOX Red, JC-1, and DMSO were purchased from Thermo Fisher Scientific (Waltham, MA). Ionomycin was purchased from LKT Laboratories (St. Paul, MN). All other reagents were purchased from Sigma-Aldrich (St. Louis, MO).

## 3. Results

### 3.1 Correlation between mitochondrial superoxide production and cytosolic calcium responses

**Fig 1:**
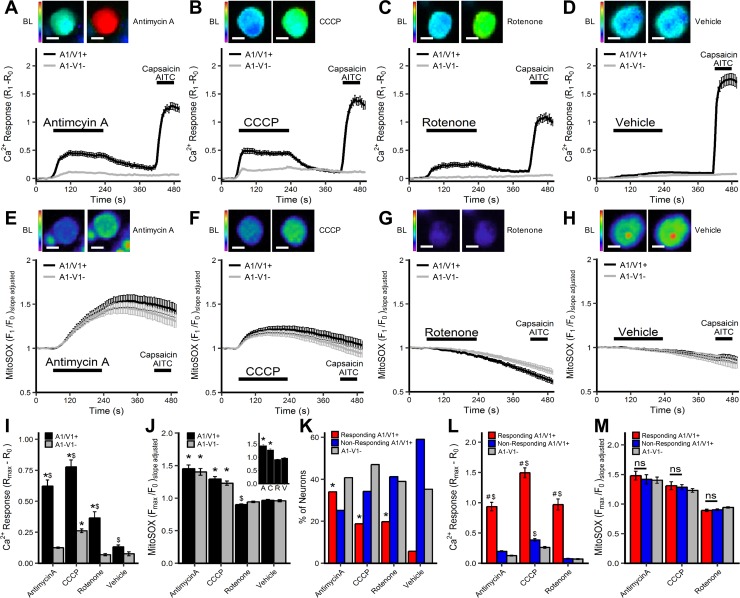
[Ca^2+^]_i_ and mitochondrial superoxide responses of vagal neurons to antimycin A, CCCP, rotenone and vehicle. Mean +/- SEM [Ca^2+^]_i_ responses (*A–D*) and mitochondrial superoxide (MitoSOX) responses (*E–H*) in A1/V1+ (black line) and A1-V1- (grey line) neurons treated with 10μM antimycin A (*A*, *E*), 10μM CCCP (*B*, *F*), 5μM rotenone (*C*, *G*) and 0.1% DMSO (*D*, *H*). Each panel includes representative Fura-2AM/MitoSOX image of a A1/V1+ neuron before and after treatment with the mitochondrial agent. A1/V1+ neurons are defined by increase in [Ca^2+^]_i_ following AITC/Caps treatment. (*I*) Mean +/- SEM maximal [Ca^2+^]_i_ response of A1/V1+ and A1-V1- neurons. (*J*) Mean +/- SEM maximal mitochondrial superoxide response of A1/V1+ and A1-V1- neurons (*insert*, mean +/- SEM maximal mitochondrial superoxide response of all neurons). (*K*) Percentage of neurons defined as ‘responding’ A1/V1+ neurons, ‘non-responding’ A1/V1+ neurons and A1-V1- neurons. (*L*) Mean +/- SEM maximal [Ca^2+^]_i_ response of ‘responding’ A1/V1+ neurons, ‘non-responding’ A1/V1+ neurons and A1-V1- neurons. (*M*) Mean +/- SEM maximal mitochondrial superoxide response of ‘responding’ A1/V1+ neurons, ‘non-responding’ A1/V1+ neurons and A1-V1- neurons. * denotes significant difference compared to vehicle (p<0.05), $ denotes significant difference between A1/V1+ and A1-V1- groups (p<0.05), # denotes significant difference between ‘responding’ and ‘non-responding’ A1/V1+ groups (p<0.05), ns denotes non-significance (p>0.05).

Endogenous mediators modulate mitochondria at several different sites, thus we investigated neuronal responses to three mitochondrial agents: antimycin A (Complex III inhibitor), CCCP (uncoupling agent) and rotenone (Complex I inhibitor). We evaluated the contribution of mitochondrial superoxide production to neuronal activation by simultaneously measuring mitochondrial superoxide (MitoSOX Red) and cytosolic calcium (Fura-2AM) in dissociated vagal neurons using live cell co-imaging in response to antimycin A (10μM, n = 282 from 8 experiments), CCCP (10μM, n = 266 from 10 experiments), rotenone (5μM, n = 177 from 6 experiments) or DMSO vehicle (0.1%, n = 122 from 7 experiments). Approximately 60% of vagal sensory neurons were defined as A1/V1+, i.e. they subsequently responded to a combined treatment of 1μM capsaicin and 100μM AITC. All three agents produced a substantially larger increase in [Ca^2+^]_i_ in the A1/V1+ neuronal population compared to the A1-V1- population (p<0.05)(Fig 1A, 1B, 1C and 1I). Indeed the vehicle also produced a minor, yet significant, increase in [Ca^2+^]_i_ in A1/V1+ neurons versus A1-V1- neurons (p<0.05)(Fig 1D and 1I). Nevertheless the increase in [Ca^2+^]_i_ in A1/V1+ neurons in response to antimycin A, CCCP and rotenone was significantly greater than that evoked by vehicle (p<0.05)(Fig 1I). This data is consistent with our previous studies demonstrating antimycin A evoked calcium transients that were dependent on extracellular calcium and TRPA1 and TRPV1 [28]. Only CCCP evoked an increase in [Ca^2+^]_i_ in A1-V1- neurons that was greater than vehicle (p<0.05)(Fig 1B, 1D and 1I).

MitoSOX measurements in the dual imaging studies showed that antimycin A and CCCP evoked significant mitochondrial superoxide production compared to vehicle in A1/V1+ neurons, A1-V1- neurons and the total neuronal population (p<0.05)(Fig 1E, 1F, 1H and 1J) (p<0.05). Despite the differences in calcium responses between A1/V1+ and A1-V1- populations, there was no differences in the magnitude of mitochondrial superoxide production in A1/V1+ and A1-V1- neurons evoked by either antimycin A or CCCP (p>0.05)(Fig 1E, 1F and 1J). Rotenone failed to evoke significant mitochondrial superoxide production in either A1/V1+, A1-V1- or total populations compared to vehicle (Fig 1G, 1H and 1J). In general, rotenone had a minor inhibitory effect on the accumulation of MitoSOX fluorescence, which was more pronounced in the A1/V1+ population compared to the A1-V1- population (p<0.05)(Fig 1G and 1J).

When we evaluated the calcium responses on a cell-by-cell basis we found that the mitochondrial agents only increased [Ca^2+^]_i_ beyond that evoked in A1-V1- neurons in a proportion of A1/V1+ neurons (see methods for definition of the threshold): antimycin A: 96/167, CCCP: 50/141, rotenone: 35/108 (Fig 1K). This ‘responding’ A1/V1+ population represents the A1/V1+ neurons ‘activated’ by the mitochondrial modulators and, consistent with this, the increase in [Ca^2+^]_i_ evoked by antimycin A, CCCP and rotenone was significantly greater in the ‘responding’ A1/V1+ population than either the ‘non-responding’ A1/V1+ or A1-V1- populations (p<0.05)(Fig 1L). CCCP-evoked increases in [Ca^2+^]_i_ were marginally yet significantly greater in ‘non-responding’ A1/V1+ compared to A1-V1- neurons (p<0.05), but this was not the case for either antimycin A or rotenone (p>0.05)(Fig 1L). Importantly, there was no difference in mitochondrial superoxide production between ‘responding’ and ‘non-responding’ A1/V1+ neurons or A1-V1- neurons for any of the mitochondrial modulators (p>0.05) (Fig 1M).

**Fig 2:**
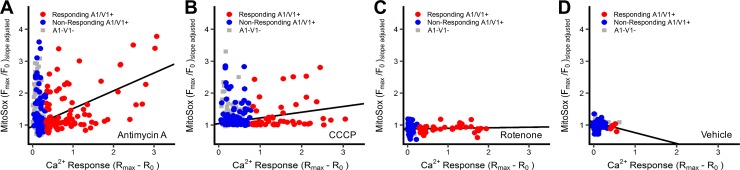
Mitochondrial superoxide responses correlates with [Ca^2+^]_i_ responses for antimycin A, but not CCCP or rotenone. Co-imaging of [Ca^2+^]_i_ and superoxide in neurons treated with 10μM antimycin A (*A*), 10μM CCCP (*B*), 5μM rotenone (*C*) and 0.1% DMSO (*D*). Trendlines depict the correlation in ‘responding’ A1/V1+ neurons. Slope and significance of each trendline is presented in Table 1.

Scatterplot analysis of the calcium and MitoSOX responses (Fig 2) showed that mitochondrial superoxide production in response to antimycin A and CCCP was highly variable in all neuronal populations (i.e. ‘responding’ A1/V1+, ‘non-responding’ A1/V1+, and A1-V1- groups). Overall, only 40% and 23% of neurons exhibited an increase in mitochondrial superoxide production following treatment with antimycin A and CCCP, respectively (compared to 1.6% for vehicle, see methods for calculation). Antimycin A-evoked calcium responses correlated with mitochondrial superoxide production in the responding A1/V1+ population and in the total A1/V1+ population (p<0.05)(Fig 2A, Table 1). However, there was no correlation between calcium and MitoSOX responses for CCCP in any neuronal population (p>0.05)(Fig 2B, Table 1). Rotenone failed to evoke mitochondrial superoxide production in any neuronal population (0%) and as such there was no correlation between calcium and MitoSOX responses (p>0.05)(Fig 2C, Table 1).

**Table 1:** Trendlines for the correlation of [Ca^2+^]_i_ and mitochondrial superoxide in subsets of vagal A1/V1+ neurons after treatment with antimycin A, CCCP and rotenone.

	Responding A1/V1+ neurons			Non-Responding A1/V1+ neurons			All A1/V1+ neurons		
	Slope	p	Sig.	Slope	p	Sig.	Slope	p	Sig.
**Wild-type**									
Antimycin A	0.48	3.03E-08	*	-0.04	0.99		0.31	2.68E-06	*
CCCP	0.27	0.06		-0.03	0.72		0.13	0.15	
Rotenone	0.41	0.31		-0.02	0.71		-0.06	0.55	
Vehicle	-0.15	0.69		0.10	0.13		0.00	0.51	
**TRPV1-/-**									
Antimycin A	0.04	0.23		-0.01	0.84		0.05	0.10	
**TRPA1-/-**									
Antimycin A	0.42	0.03	*	-0.01	0.95		-0.04	-0.07	

*: Denotes p<0.05

**Fig 3:**
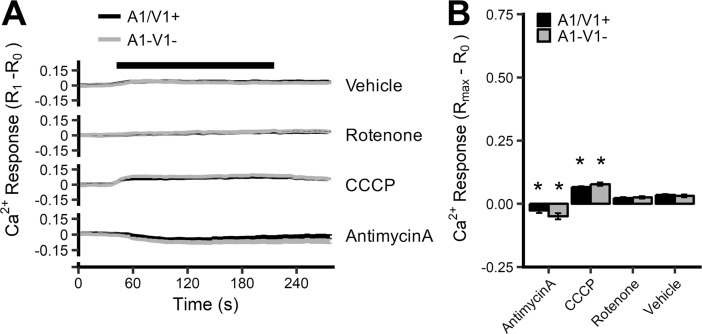
[Ca^2+^]_i_ responses of vagal neurons to antimycin A, CCCP, rotenone and vehicle in the nominal absence of extracellular calcium. (*A*) Mean +/- SEM [Ca^2+^]_i_ responses in A1/V1+ (black line) and A1-V1- (grey line) neurons treated with 10μM antimycin A, 10μM CCCP, 5μM rotenone and 0.1% DMSO. Blocked line denotes stimulus application. A1/V1+ neurons are defined by increase in [Ca^2+^]_i_ following AITC/Caps treatment in the presence of 2.5mM CaCl_2_ (data not shown). (*B*) Mean +/- SEM maximal [Ca^2+^]_i_ response of A1/V1+ and A1-V1- neurons. * denotes significant difference compared to vehicle (p<0.05), $ denotes significant difference between A1/V1+ and A1-V1- groups (p<0.05).

We have shown previously that antimycin A-induced calcium responses in nociceptive neurons were largely dependent on calcium influx through the cation channels TRPA1 and TRPV1 [28]. Here, we found in a separate series of Fura-2AM studies in vagal neurons (n = 381) that increases in [Ca^2+^]_i_ in A1/V1+ neurons in response mitochondrial modulation were largely dependent on extracellular calcium (Fig 3). There were no differences in calcium responses in A1/V1+ and A1-V1- neurons in response to antimycin A (10μM), CCCP (10μM), rotenone (5μM) or DMSO vehicle (0.1%) when extracellular calcium was buffered to 75nM with EGTA (p>0.05). Only CCCP evoked significantly greater responses in vagal neurons compared to vehicle (p<0.05), suggesting that some of the CCCP-evoked calcium transient (in both A1/V1+ and A1-V1- neurons) was dependent on intracellular stores.

**Fig 4:**
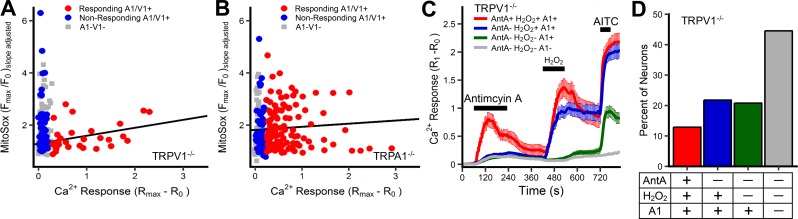
Antimycin A-induced [Ca^2+^]_i_ responses correlates with mitochondrial superoxide responses in trpv1^-/-^ A1+ neurons but not trpa1^-/-^ V1+ neurons. Co-imaging of [Ca^2+^]_i_ and superoxide in neurons treated with 10μM antimycin A in neurons from TRPV1^-/-^ (*A*) and TRPA1^-/-^ (*B*) mice. Trendlines depict the correlation in ‘responding’ neurons. Slope and significance of each trendline is presented in Table 1. (*C*) Mean +/- SEM [Ca^2+^]_i_ responses in neurons from TRPV1^-/-^ mice treated with 10μM antimycin A, 300μM H_2_O_2_ and 100μM AITC. Neurons allocated to groups based upon sensitivity to the stimuli. A1+ defined by response to AITC. (*D*) Percentage of neurons from TRPV1^-/-^ mice allocated to groups shown in *C*.

TRPA1 is directly activated by ROS [14–16], whereas there is little evidence that TRPV1 is activated by ROS [14,16,35]. We therefore investigated antimycin A-evoked Fura-2AM calcium and MitoSOX responses in vagal neurons from TRPA1^-/-^ and TRPV1^-/-^ mice (n = 258 from 9 experiments and n = 201 from 10 experiments, respectively). [Ca^2+^]_i_ and mitochondrial superoxide production correlated in ‘responding’ trpv1^-/-^ A1+ neurons (p<0.05)(Fig 4A, Table 1) but not in ‘responding’ trpa1^-/-^ V1+ neurons (p>0.05)(Fig 4B, Table 1). Similar to the wild-type A1/V1+ neurons, mitochondrial modulation with antimycin A failed to evoke calcium responses in all trpv1^-/-^ A1+ neurons, suggesting the possibility that some neurons expressing the TRPA1 channel were insensitive to ROS. Sequential exposure of vagal neurons from TRPV1^-/-^ mice (n = 202 from 7 experiments) to antimycin A (10μM), H_2_O_2_ (300μM) and then AITC (100μM), showed that a significant proportion of antimycin-insensitive, A1+ neurons did indeed respond to the H_2_O_2_ with an increase in calcium (Fig 4C and 4D). This data confirms the sensitivity of TRPA1 channels in these neurons to exogenously applied ROS H_2_O_2_.

### 3.2 Correlation between mitochondrial depolarization and cytosolic calcium responses

Given that our data suggests that mitochondrial superoxide is not the only determinant of mitochondrial modulation-induced A1/V1+ neuronal activation, we next investigated mitochondrial depolarization–another hallmark of mitochondrial dysfunction. We performed live cell co-imaging of cytosolic calcium (Fura-2AM) and mitochondrial polarization (JC-1) during treatment with antimycin A (10μM, n = 90 from 6 experiments), CCCP (10μM, n = 210 from 10 experiments), rotenone (5μM, n = 112 from 7 experiments) and DMSO vehicle (0.1%, n = 129 from 8 experiments).

**Fig 5:**
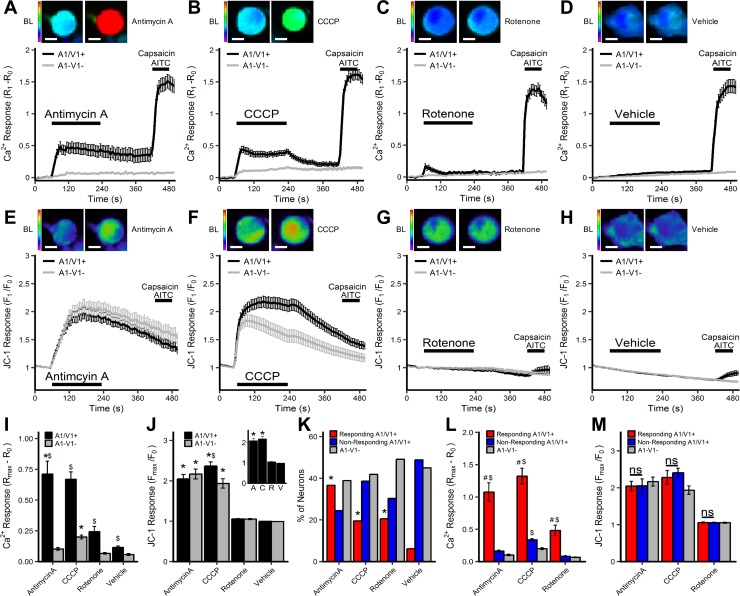
[Ca^2+^]_i_ and mitochondrial polarization responses of vagal neurons to antimycin A, CCCP, rotenone and vehicle. Mean +/- SEM [Ca^2+^]_i_ responses (*A–D*) and mitochondrial polarization (JC-1) responses (*E–H*) in A1/V1+ (black line) and A1-V1- (grey line) neurons treated with 10μM antimycin A (*A*, *E*), 10μM CCCP (*B*, *F*), 5μM rotenone (*C*, *G*) and 0.1% DMSO (*D*, *H*). Each panel includes representative Fura-2AM/JC-1 image of an A1/V1+ neuron before and after treatment with the mitochondrial agent. A1/V1+ neurons are defined by increase in [Ca^2+^]_i_ following AITC/Caps treatment. (*I*) Mean +/- SEM maximal [Ca^2+^]_i_ response of A1/V1+ and A1-V1- neurons. (*J*) Mean +/- SEM maximal mitochondrial depolarization of A1/V1+ and A1-V1- neurons (*insert*, mean +/- SEM maximal mitochondrial depolarization of all neurons). (*K*) Percentage of neurons defined as ‘responding’ A1/V1+, ‘non-responding’ A1/V1+ and A1-V1-. (*L*) Mean +/- SEM maximal [Ca^2+^]_i_ response of ‘responding’ A1/V1+, ‘non-responding’ A1/V1+ and A1-V1-. (*M*) Mean +/- SEM maximal mitochondrial depolarization of ‘responding’ A1/V1+, ‘non-responding’ A1/V1+ and A1-V1-. * denotes significant difference compared to vehicle (p<0.05), $ denotes significant difference between A1/V1+ and A1-V1- groups (p<0.05), # denotes significant difference between ‘responding’ and ‘non-responding’ A1/V1+ groups (p<0.05), ns denotes non-significance (p>0.05).

Again, approximately 60% of vagal sensory neurons were defined as A1/V1+, and all three mitochondrial agents evoked substantially larger increases in [Ca^2+^]_i_ in the A1/V1+ neuronal population compared to the A1-V1- population (p<0.05)(Fig 5A, 5B, 5C and 5I), and these responses were significantly greater than those evoked by vehicle (p<0.05)(Fig 5D and 5I). Again, CCCP alone evoked an increase in [Ca^2+^]_i_ in A1-V1- neurons that was greater than vehicle (p<0.05)(Fig 5B, 5D and 5I). Antimycin A and CCCP both caused significant mitochondrial depolarization compared to vehicle (p<0.05) but there were no differences between A1/V1+ and A1-V1- populations (p>0.05)(Fig 5E, 5F, 5H and 5J). Mitochondrial depolarization occurred in 97% and 80% of neurons in response to antimycin A and CCCP, respectively (compared to 0.8% for vehicle). Although rotenone failed to evoke significant mitochondrial depolarization compared to vehicle in the total, A1/V1+ and A1-V1- populations overall (Fig 5G and 5J), rotenone did evoke mitochondrial depolarization in 38% of neurons (compared to 0.8% for vehicle).

**Fig 6:**
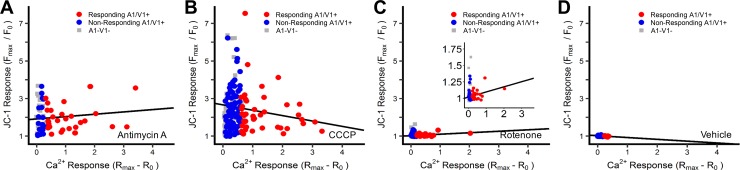
Mitochondrial depolarization responses correlates with [Ca^2+^]_i_ responses for rotenone, but not antimycin A or CCCP. Co-imaging of [Ca^2+^]_i_ and mitochondrial polarization in neurons treated with 10μM antimycin A (*A*), 10μM CCCP (*B*), 5μM rotenone (*C*) and 0.1% DMSO (*D*). Trendlines depict the correlation in ‘responding’ A1/V1+ neurons. Slope and significance of each trendline is presented in Table 2.

Analysis of individual neuronal responses showed again that the mitochondrial agents only increased [Ca^2+^]_i_ beyond that evoked in A1-V1- neurons in a proportion of A1/V1+ neurons: antimycin A: 33/55, CCCP: 41/122, rotenone: 23/57 (Fig 5K). Indeed, the increase in [Ca^2+^]_i_ evoked by antimycin A, CCCP and rotenone was significantly greater in the ‘responding’ A1/V1+ population than either the ‘non-responding’ A1/V1+ or A1-V1- populations (p<0.05)(Fig 5L). Notably, there were no differences in mitochondrial depolarization between ‘responding’ A1/V1+ and ‘non-responding’ A1/V1+ neurons for each of the agents (p>0.05)(Fig 5M). Using scatterplots of the cell-by-cell responses, we found there was no correlation of calcium responses evoked by either antimycin A or CCCP to mitochondrial depolarization in ‘responding’ A1/V1+ neurons (p>0.05)(Fig 6A and 6B, Table 2). Nevertheless, there was a minor, yet significant correlation of rotenone-induced calcium responses to mitochondrial depolarization in ‘responding’ A1/V1+ neurons (p>0.05)(Fig 6C, Table 2), consistent with the finding that some neurons exhibited mitochondrial depolarization with rotenone.

**Table 2:** Trendlines for the correlation of [Ca^2+^]_i_ and mitochondrial depolarization in subsets of vagal A1/V1+ neurons after treatment with antimycin A, CCCP and rotenone.

	Responding A1/V1+ neurons			Non-Responding A1/V1+ neurons			All A1/V1+ neurons		
	Slope	p	Sig.	Slope	p	Sig.	Slope	p	Sig.
**Wild-type**									
Antimycin A	0.15	0.22		0.02	0.13		0.09	0.26	
CCCP	-0.12	0.88		0.03	0.03	*	-0.05	0.82	
Rotenone	2.08	0.03	*	-0.12	0.87		0.71	0.09	
Vehicle	-0.45	0.69		0.13	0.37		-0.48	0.81	

*: Denotes p<0.05

## 4. Discussion

Nociceptive activation is a hallmark of inflammation in multiple organs, resulting in debilitating symptoms (e.g. pain) and reflexes (e.g. vasodilation, edema, bronchospasm, etc.) [36–38]. While inflammatory signaling pathways involving TNFα, ceramides and neurotrophins inhibit complexes of the mETC and induce mitochondrial dysfunction (ROS production, depolarization, and calcium release) [24–27], the mechanisms linking these factors to C-fiber activity are largely unknown. In a previous study, we demonstrated that inhibition of mETC complex III with antimycin A activated bronchopulmonary C-fibers via the activation of TRPA1 and TRPV1 [28]–Ca^2+^-permeable cation channels selectively expressed on many nociceptive sensory neurons [7,28,39]. However, the influence of specific aspects of mitochondrial function on TRPA1 and TRPV1 activation remains unknown. Here, we investigated the correlation of mitochondrial depolarization and superoxide production on calcium fluxes in dissociated vagal neurons following mitochondrial modulation.

As inflammatory signaling inhibits the mETC at multiple sites, we performed these experiments using three mitochondrial agents: antimycin A (complex III inhibitor), rotenone (complex I inhibitor), and CCCP (mitochondrial uncoupling agent). Here, we found that all three agents caused robust increases in [Ca^2+^]_i_ in the vagal A1/V1+ neuronal population. These data are consistent with our previous study that antimycin A evoked a selective increase in [Ca^2+^]_i_ in A1/V1+ vagal neurons that was abolished by the removal of extracellular Ca^2+^ or by the genetic and pharmacological blockade of the nociceptive-specific ion channels TRPA1 and TRPV1 [28]. Given that TRPA1/TRPV1 were required for antimycin A-induced bronchopulmonary C-fiber activation [28], it is likely that the mitochondrial agent-evoked increases in [Ca^2+^]_i_ in A1/V1+ neurons observed in this study represents preferential activation of A1/V1+ neurons. However, not all A1/V1+ neurons ‘responded’ to mitochondrial modulation: approximately 60%, 35% and 35% of A1/V1+ neurons exhibited an increase in [Ca^2+^]_i_ to antimycin A, CCCP and rotenone, respectively. In general, antimycin A and CCCP evoked greater increases in [Ca^2+^]_i_ in ‘responding’ A1/V1+ neurons than rotenone. There was no correlation between responses in individual A1/V1+ neurons and their proximity to other neurons (data not shown), suggesting that calcium responses in each neuron was independent of the effects of mitochondrial modulation in other neurons.

Mitochondrial modulation can evoke ROS production, most notably at complexes I and III, when electrons leak and incompletely oxidize O_2_ resulting in O_2_^•-^ (superoxide) formation [40–43]. Superoxide is membrane impermeable and highly reactive, thus is capable of damaging DNA, proteins and lipids. In cells, superoxide dismutase rapidly converts superoxide to H_2_O_2_ which is membrane permeable but less reactive [44]. Here, we found that antimycin A increased mitochondrial superoxide production acutely in both A1/V1+ and A1-V1- neurons. Furthermore, there was no difference in the mitochondrial superoxide production evoked by antimycin A between the ‘responding’ A1/V1+ and ‘non-responding’ neurons. Nevertheless, there was a significant correlation between antimycin A-evoked mitochondrial superoxide production and the magnitude of calcium responses in ‘responding’ A1/V1+ neurons. Our interpretation of these data is that mitochondrial superoxide produced by antimycin A can lead to A1/V1+ neuronal activation, but this is dependent on another unknown factor that is not present in all A1/V1+ neurons. It should be noted that only 40% of all neurons exhibited increased superoxide production in response to antimycin A, raising the possibility that some neurons are insensitive to this mitochondrial modulating agent. However, this seems unlikely given the observation that 97% of neurons exhibited antimycin A-induced mitochondrial depolarization.

Both superoxide and H_2_O_2_ are potent activators of TRPA1 [15,16]. Here, we found a significant correlation between antimycin A-evoked mitochondrial superoxide production and the magnitude of calcium responses in ‘responding’ trpv1^-/-^ A1+ neurons, whose responses are presumed to be mediated by ROS-sensitive TRPA1 alone [28]. Nevertheless, some trpv1^-/-^ A1+ neurons failed to be activated despite substantial superoxide production, thus again suggesting that some other unknown factor is required for mitochondrial ROS-mediated TRPA1 activation. Separate studies confirmed that many non-responding trpv1^-/-^ A1+ neurons were activated by exogenously-applied H_2_O_2_. Importantly, there was no correlation between antimycin A-evoked mitochondrial superoxide production and the magnitude of calcium responses in ‘responding’ trpa1^-/-^ V1+ neurons, whose responses are likely mediated by TRPV1 [28]. There is little evidence to suggest that TRPV1 is a major target of ROS and oxidative stress: TRPV1 is not activated by physiological concentrations of H_2_O_2_ [16,35] or most electrophilic products of lipid peroxidation [45], although it is activated by high concentrations of 4-oxononenal [11]. Instead, TRPV1 is activated by PIP_2_ hydrolysis, GPCR signaling and arachidonic acid metabolites [45–48]. Our data further suggests that TRPV1-mediated activation by antimycin A is not downstream of superoxide production.

Like antimycin A, CCCP evoked significant mitochondrial superoxide production in all neuronal populations, consistent with previous reports in isolated rat brain mitochondria [49], although other studies indicate that uncoupling agents such as CCCP do not increase ROS production [50,51]. However, there was no correlation between CCCP-induced mitochondrial superoxide production and the magnitude of calcium responses in ‘responding’ A1/V1+ neurons. Approximately 80% of neurons exhibited mitochondrial depolarization to CCCP, but there were no significant differences between the mitochondrial depolarization in ‘responding’ A1/V1+ and ‘non-responding’ neurons and there was no correlation between mitochondrial depolarization and the magnitude of calcium responses in ‘responding’ A1/V1+ neurons. We therefore conclude that factors other than mitochondrial superoxide production determine the CCCP-induced activation of A1/V1+ neurons and that mitochondrial depolarization alone is unable to account for these responses.

Rotenone failed to cause mitochondrial superoxide production in any neuronal population. Indeed, rotenone decreased baseline mitochondrial ROS production in A1/V1+ neurons. Previous studies have shown that rotenone can increase or decrease ROS production depending on metabolism of complex I versus complex II substrates [42,52]. Despite the lack of superoxide production, rotenone evoked an increase in [Ca^2+^]_i_ in approximately 35% of A1/V1+ neurons in this study. Although rotenone caused mitochondrial depolarization in approximately 38% of all vagal neurons, there were no significant differences between the mitochondrial depolarization in ‘responding’ A1/V1+ and ‘non-responding’ neurons. Nevertheless, there was a significant correlation between rotenone-evoked mitochondrial depolarization and the magnitude of calcium responses in ‘responding’ A1/V1+ neurons. This suggests that mitochondrial depolarization alone can lead to A1/V1+ neuronal activation, but again this is dependent on another unknown factor that is not present in all A1/V1+ neurons.

Our data indicates that mitochondrial modulators (in particular antimycin A and CCCP) evoke mitochondrial superoxide production in a limited percentage of neurons despite the evident mitochondrial depolarization in the majority of neurons. The present study used quantitative measurements of mitochondrial superoxide using MitoSOX, which is neither ratiometric nor reversible, making it possibly susceptible, despite our efforts to normalize the data, to variations in loading and baseline redox state. MitoSOX is targeted to the mitochondrial matrix, thus it is possible that some of the superoxide produced from complex I and III may evade MitoSOX detection following its release into the intermembrane space [41,53,54]. ROS produced within sensory neurons has 3 potential fates: reacting with signaling pathways, with endogenous antioxidant systems (e.g. glutathione) or with the ROS-sensitive dye. As such, the quantification of ROS production may be susceptible to significant error on a cell-by-cell basis. Nevertheless, even a qualitative analysis suggests that (A) mitochondrial modulation does not evoke significant calcium responses in all A1/V1+ neurons and (B) significant ROS production does not necessarily evoke calcium responses in A1/V1+ neurons. This suggests that factors in addition to superoxide determine the TRPA1- and TRPV1-mediated calcium responses in A1/V1+ neurons. The identity of these factors is presently unknown, but it is possible that these either involve the activity of antioxidant systems or the spatial/temporal arrangement of mitochondria, TRP channels and signaling molecules. Our rotenone studies suggest that mitochondrial depolarization is able to contribute, but experiments with CCCP (and antimycin A) indicate that mitochondrial depolarization is not guaranteed to result in significant calcium responses in A1/V1+ neurons. How mitochondrial depolarization could contribute to A1/V1+ neuronal activation is not yet known. Mitochondrial depolarization causes calcium efflux from the matrix, and calcium is a regulator of multiple pathways such as phospholipases and protein kinases [55,56] that can alter TRP channel activity [45,57–59].

Unlike antimycin A and rotenone, CCCP evoked a significant increase in [Ca^2+^]_i_ in A1-V1- neurons, although these were much smaller than those in A1/V1+ neurons. As such, the threshold for determining the calcium ‘responsiveness’ of a particular neurons is greater for CCCP treatments. A1-V1- neurons do not express TRPA1 or TRPV1. Thus, the source of the CCCP-evoked calcium responses in A1-V1- is presently unknown. It is possible that calcium release from the mitochondrial matrix after robust mitochondrial depolarization contributes to these calcium transients [60], and this is consistent with (A) the significant calcium responses with CCCP in the nominal absence of extracellular calcium and (B) the significant correlation between CCCP-evoked mitochondrial depolarization and the magnitude of calcium responses in A1-V1- neurons (slope 0.035, p<0.05, data not shown).

The present study has investigated the correlation between mitochondrial ROS/depolarization and calcium transients evoked by mitochondrial modulators in vagal neurons. Our previous study identified the critical role of TRPA1 and TRPV1 in antimycin A-induced calcium responses and bronchopulmonary C-fiber activation [28]. Although the specific role of TRPA1 and TRPV1 has not been extensively investigated in the present study, our data is consistent with TRPA1 and TRPV1 mediating the mitochondrial modulator-evoked calcium responses. It is important to note, however, that TRP channel sensitivity itself is not static and that multiple intracellular pathways, including oxidative stress, can promote either sensitization or desensitization of TRPA1 and TRPV1 channels [3,61–64]. Furthermore, TRPA1 and TRPV1 have been reported to functionally modulate each other’s sensitivity [65,66]. Although not addressed here, it is possible that mitochondrial modulation may impact TRP function beyond channel activation.

In summary, we have shown that mitochondrial modulation by antimycin A-induced TRPA1-mediated calcium responses in vagal A1/V1+ neurons correlates with mitochondrial superoxide production, but that other factors including mitochondrial depolarization may contribute to A1/V1+ neuronal calcium responses via TRPV1 and downstream of complex I inhibition and mitochondrial uncoupling. It should be emphasized that the conclusions here are based upon correlations, which do not necessarily imply causation. Thus, we note that these studies do not show a conclusive role of superoxide production in the A1/V1+ neuronal calcium responses by mitochondrial modulators. Furthermore, we have focused our present study on dissociated vagal neurons and it is possible that mechanisms linking mitochondrial superoxide production and depolarization with TRP channel activation may be different within sensory terminals in vivo. More studies are needed to fully understand the events linking mitochondrial dysfunction and nociceptor activation.

## Supporting information

S1 Dataset Raw data for dual imaging of Fura2 and mitoSOX. (XLSX)

S2 Dataset Raw data for dual imaging of Fura2 and JC-1. (XLSX)

## Abbreviations

AITC: allyl isothiocyanate
[Ca^2+^]_i_: intracellular calcium concentration)
CCCP: carbonyl cyanide m-chlorophenyl hydrazine
JC-1: 5,5′,6,6′-Tetrachloro-1,1′,3,3′-tetraethyl-imidacarbocyanine iodide
mETC: mitochondrial electron transport chain
ROS: reactive oxygen species
TRPA1: transient receptor potential ankyrin 1
TRPV1: transient receptor potential vanilloid 1
